# Effectiveness of Gabapentinoids in Neuropathic Pain: A Single-Center Retrospective Study at a Specialized Institution in Mexico

**DOI:** 10.3390/pharmacy14020055

**Published:** 2026-03-29

**Authors:** Carlos Eduardo Estrada-De La Rosa, Felipe Alexis Avalos-Salgado, Nancy Evelyn Navarro-Ruiz, Erika Fabiola López-Villalobos, Roberto de Jesús Sandoval-Muñiz, Monserratt Abud-González, María Luisa Muñoz-Almaguer, Raymundo Escutia-Gutiérrez

**Affiliations:** 1 Instituto Jalisciense de Alivio al Dolor y Cuidados Paliativos, Secretaria de Salud Jalisco, Zapopan 45150, Mexico; carlos.estrada@red.jalisco.gob.mx; 2 Departamento de Farmacobiología, Centro Universitario de Ciencias Exactas e Ingenierías (CUCEI), Universidad de Guadalajara, Guadalajara 44430, Mexico; alexis.avalos@academicos.udg.mx (F.A.A.-S.); monserratt.abud@academicos.udg.mx (M.A.-G.); maria.malmaguer@academicos.udg.mx (M.L.M.-A.); 3 Departamento de Vida Saludable y Promoción de la Salud, Centro Universitario de Tlajomulco, Universidad de Guadalajara, Tlajomulco de Zuñiga 45641, Mexico; nancye.navarro@academicos.udg.mx; 4 Laboratorio de Análisis Clínicos e Investigación Traslacional, Centro Universitario de Ciencias Exactas e Ingenierías (CUCEI), Universidad de Guadalajara, Guadalajara 44430, Mexico; erikafabiola.lopez@academicos.udg.mx; 5 Programa de Capacitación Región Sanitaria XI Centro-Tonalá, Secretaría de Salud Jalisco, Guadalajara 44730, Mexico; roberto.sandovalm.rsh@gmail.com

**Keywords:** neuropathic pain, gabapentinoids, medication safety, pain management, adverse drug reactions, Mexico

## Abstract

Background/Objectives: Gabapentinoids are first-line treatments for neuropathic pain (NP); however, real-world evidence regarding their safety and effectiveness in complex clinical populations remains limited. This study aimed to evaluate the effectiveness and safety profile of gabapentinoid therapy in patients managed within a specialized pain relief institution. Methods: A retrospective cohort study (*n* = 109) was conducted (January 2024 to December 2024). Effectiveness was assessed via DN4 and VAS over one year. Time to improvement was analyzed using Kaplan–Meier curves. Results: The cohort (mean age 66.2 ± 15.3 years) presented 100% comorbidity and polypharmacy (66.1% opioids; 67.9% antidepressants). Although all patients showed improvement, only 35.8% achieved “maximal improvement.” Pregabalin demonstrated faster VAS reduction than gabapentin (*p* = 0.029), though long-term success was comparable (*p* = 0.30). Significantly, 100% of patients reported at least one adverse drug event (ADE), primarily somnolence (66.1%), though no serious ADEs occurred. Lower baseline pain scores were significant predictors of therapeutic success. Conclusions: Gabapentinoids are effective for long-term NP management; however, their use is consistently associated with non-serious ADEs. In specialized settings characterized by extensive CNS-active polypharmacy, proactive pharmacovigilance and multidisciplinary oversight are essential to balance analgesic effectiveness with medication safety.

## 1. Introduction

Neuropathic pain (NP) is defined as pain caused by a lesion or disease of the somatosensory nervous system [1]. This condition is etiologically classified into peripheral neuropathic pain—arising from damage to peripheral nerves, dorsal root ganglia, or dorsal roots—and central neuropathic pain, resulting from injuries to the spinal cord or brain [2]. Among peripheral manifestations, diabetic peripheral neuropathy (DPN) is particularly prevalent, characterized by symmetrical sensory symptoms such as numbness and burning pain [3]. Unlike nociceptive pain, NP often lacks a protective biological function and imposes a significant burden of morbidity, profoundly affecting quality of life and requiring specialized diagnostic and therapeutic approaches [4]. In this context, gabapentinoids—primarily gabapentin and pregabalin—have become established mainstays of pharmacological management. These agents exert their effects by binding to the α2δ subunit of voltage-gated calcium channels, modulating the release of excitatory neurotransmitters [5]. While they are considered first-line treatment options according to international clinical guidelines [6,7,8,9], their widespread use in complex, real-world populations raises critical questions regarding medication safety. Beyond efficacy, gabapentinoids are associated with various adverse drug events (ADEs), including dizziness, somnolence, and severe risks such as respiratory depression or potential misuse. These risks are particularly relevant for patients with multiple comorbidities or those on polypharmacy regimens. From a systems approach to medication safety, a notable gap exists between the controlled environment of Randomized Controlled Trials (RCTs) and routine clinical practice. RCTs often exclude the very patients most vulnerable to safety issues, the elderly, those with renal impairment, or those taking interacting medications. Consequently, real-world evidence and pharmacovigilance studies are essential to validate not only the effectiveness but also the safety profile of these treatments in everyday settings. Specialized pain relief institutions represent an innovation in pharmacy practice, providing a structured environment in which pharmacological interventions can be closely monitored to mitigate risks. Therefore, this study aimed to evaluate the effectiveness and safety-related outcomes of gabapentinoid agents in a specialized institution in Mexico. Our findings contribute to a better understanding of how systemic clinical oversight can optimize patient safety in neuropathic pain management.

## 2. Materials and Methods

### 2.1. Study Design and Setting

A retrospective cohort study was conducted at a specialized, outpatient pain management institution in Jalisco, Mexico. Data were systematically retrieved from the Electronic Health Records (EHR) of patients diagnosed with neuropathic pain (NP) who received clinical consultation between January and December 2024. This study period ensured a contemporary assessment of pharmacological interventions and safety monitoring protocols in specialized practice. The study was carried out at the Jalisco Institute for Pain Relief and Palliative Care. Patients were followed for up to 12 months post-treatment initiation. Although data collection spanned a year, outcome assessment began at the 6-month mark. This decision was made because a limited number of patients attended the 3-month follow-up visit, resulting in insufficient data for reliable statistical analysis.

### 2.2. Subject Selection

Patients were screened for inclusion based on the following eligibility criteria: Inclusion Criteria: (i) age ≥ 18 years; (ii) a confirmed clinical diagnosis of neuropathic pain; (iii) continuous treatment with a gabapentinoid agent (gabapentin or pregabalin); (iv) at least six months of clinical follow-up; and (v) documented pain intensity scores at baseline (treatment initiation) and at the end of the follow-up period. Exclusion Criteria: (i) incomplete or fragmented medical records precluding a reliable longitudinal analysis; and (ii) patients receiving palliative care at the time of inclusion, to avoid confounding variables related to end-of-life pain management. Following the screening process, 109 patients met all eligibility criteria and were included in the final analysis, while 52 patients were excluded. No a priori sample size calculation was performed, as this study included all eligible patients identified in the EHR during the study period.

### 2.3. Data Extraction

Data extraction was performed by two trained researchers using a standardized protocol to ensure inter-rater reliability. Variables were categorized into three primary domains:

- Sociodemographic and Clinical Profile: Gender, age, and type of comorbidities.
- Pain Characterization: Self-reported pain levels, specific neuropathic symptoms, and longitudinal changes over the follow-up period.
- Pharmacological and Safety Profile: Specific gabapentinoid agent prescribed, dosage regimens, concomitant medications (to assess polypharmacy), and the incidence of adverse drug events (ADEs).

### 2.4. Outcome Measures

The Douleur Neuropathique 4 (DN4) test was utilized as the primary screening and assessment tool for NP. This validated instrument consists of 10 items evaluating sensory symptoms (e.g., burning, electric shocks, tingling) and clinical signs from physical examination. A score of 4 or higher indicates a high probability of neuropathic pain [10]. Additionally, the Visual Analog Scale (VAS) was employed to quantify pain intensity, ranging from 1 (minimal/no pain) to 10 (maximal/severe pain). Both DN4 and VAS scores were recorded at baseline and during the final follow-up visit. To evaluate clinical excellence, a composite endpoint termed “Maximal Improvement” was defined as achieving a DN4 score of 1 and a VAS score of 1 or 2 in their last visit.

Information on adverse drug events was extracted retrospectively from electronic health records (EHRs), based on patient reports of negative reactions to their physician.

- Somnolence: patients report an increased sleepiness in which they have difficulty maintaining wakefulness.
- Nausea: patient reported an unpleasant sensation perceived in the throat, chest, or upper abdomen for a period of up to 30 min after drug ingestion.
- Headache: patient reported pain or discomfort localized to any region of the head.
- Confusion: patient reported a disturbance in attention, awareness, or cognition, resulting in impaired orientation, memory, or ability to think clearly.
- Generalized Weakness: patient reported a subjective reduction in overall muscular strength limiting normal physical activity.

### 2.5. Statistical Analysis

Descriptive statistics were used to summarize the cohort’s characteristics. Quantitative variables were compared using independent Student’s *t*-tests, while categorical data and proportions were analyzed using Chi-square or Fisher’s exact tests, as appropriate. A multivariate logistic regression model was used to verify association between variables, with Maximal improvement as the dependent variable. The probability of achieving clinical improvement over time was estimated using Kaplan–Meier survival curves; patients were censored due to loss to follow-up or at the end of the study period. The event of interest was defined as significant pain reduction (clinical improvement), with the observation period starting after the initial six months of gabapentinoid therapy. Patients lost to follow-up were treated as censored observations. Differences in improvement rates between subgroups were evaluated using the log-rank test. Statistical significance was set at *p* ≤ 0.05. All analyses were performed using SPSS v24 (IBM Corp., Armonk, NY, USA), and graphical figures were created using Microsoft Excel, v16.0 (Microsoft Corporation, Redmond, WA, USA).

## 3. Results

### 3.1. Sociodemographic and Clinical Baseline Characteristics

The baseline sociodemographic and clinical characteristics of the study cohort are summarized in Table 1. A total of n = 109 patients with neuropathic pain (NP) receiving gabapentinoid therapy met the eligibility criteria and were included in the final analysis. The sociodemographic profile, summarized in Table 1, revealed a female predominance (68.8%) and a mean age of 66.2 ± 15.3 years, indicating a predominantly elderly population. Regarding the etiology of NP, postherpetic neuralgia (PHN) was the most prevalent diagnosis (59.6%), followed by trigeminal neuralgia (34.9%) and diabetic peripheral polyneuropathy (5.5%). All patients presented at least one comorbidity, underscoring a high clinical complexity in this cohort. The most frequent comorbid conditions were Type 2 Diabetes Mellitus (58.7%) and Systemic Arterial Hypertension (56.0%). This high prevalence of chronic conditions highlights the presence of multimorbidity.

### 3.2. Pharmacological Profile and Baseline Pain Assessment

The pharmacological management and baseline pain intensity scores are presented in Table 2. Regarding gabapentinoid therapy, approximately one-third of the cohort (34.9%) received gabapentin, with the most frequent dosing regimen being 900 mg/day (*n* = 30), while a smaller subset (*n* = 8) was prescribed 600 mg/day. In the pregabalin group, two patients received 50 mg/day (adjusted to renal function), 22 received 75 mg/day, 18 received 150 mg/day, one received 225 mg/day, 29 received 300 mg/day, and one received 450 mg/day. At study entry, all participants (100%) met the clinical criteria for neuropathic pain according to the DN4 test (≥4). The baseline pain intensity was severe, with a mean VAS score of 8.9 ± 0.8, indicating a high symptom burden at the start of the specialized intervention.

A significant proportion of patients were receiving concomitant central nervous system (CNS) active medications, including analgesics (69.7%), antidepressants (67.9%), and opioids (66.1%). Additionally, 33.9% of the cohort utilized other antiepileptic drugs.

### 3.3. Clinical Effectiveness and Safety Outcomes

**Figure 1:**
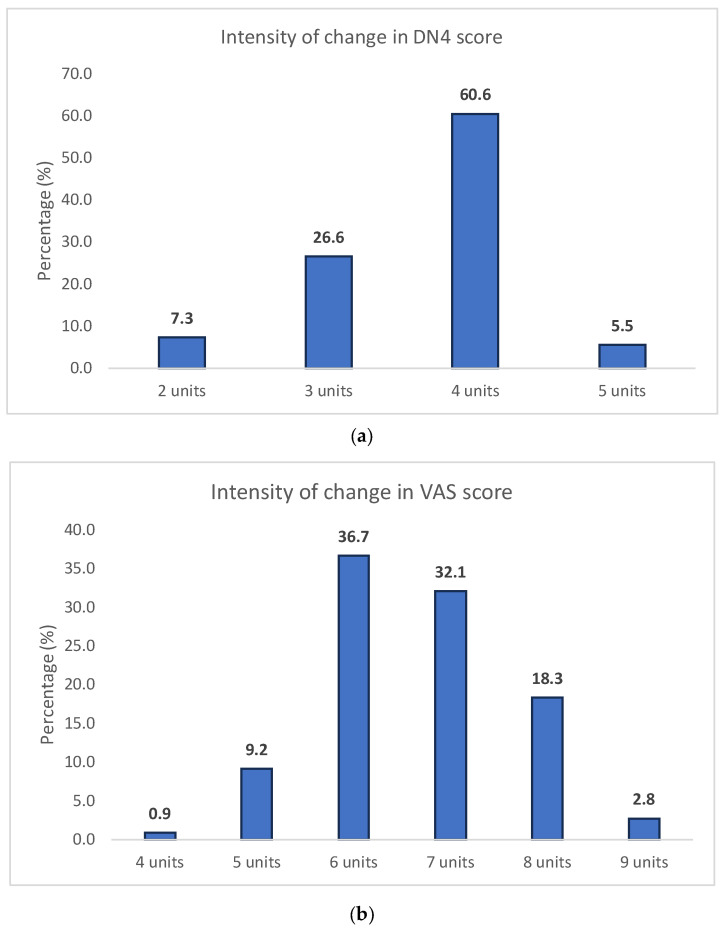
(**a**) Intensity of change in DN4 score. (**b**) Intensity of change in VAS score.

The longitudinal changes in pain intensity and the safety profile of gabapentinoid therapy are detailed in Table 3. At the final follow-up visit, all patients (100%) demonstrated a reduction in both DN4 and VAS scores compared to baseline, although intensity of improvement was not homogeneous; in Figure 1a, it is shown that 66.1% of the patients improved up to 4–5 points in DN4 score, while 33.9% saw a lower improvement of 2–3 units. Alongside this, Figure 1b shows the distribution of change in VAS score; almost all the patients improved 6 or more units, while only 10.1% improved 5 or less. However, despite this overall trend towards improvement, only one-third of the cohort achieved the predefined criteria for “Maximal Improvement” (a composite of DN4 = 1 and VAS ≤ 2). Regarding the safety profile, all participants reported at least one Adverse Drug Event (ADE) during the study period. The most prevalent ADE was somnolence (66.1%). Clinical causality assessment by the researchers determined that all reported events were associated with the pharmacological treatment; however, no Serious Adverse Events (SAEs) were recorded.

### 3.4. Comparative Analysis of Factors Associated with Maximal Improvement

To identify potential predictors of therapeutic success, the cohort was stratified into two groups: those who achieved Maximal Improvement at the final visit and those who did not. The comparative analysis, summarized in Table 4, revealed that baseline pain severity was a significant determinant of treatment outcomes. Patients who achieved maximal improvement presented with significantly lower baseline DN4 scores (4.8 ± 0.5) compared to those who did not (5.4 ± 0.7, *p* ≤ 0.001). Similarly, lower baseline VAS scores were associated with a higher probability of therapeutic success (8.5 ± 0.7 vs. 9.1 ± 0.8, *p* = 0.001). Regarding pharmacological co-interventions, a trend was observed in patients receiving concomitant antidepressant therapy, who showed a higher rate of maximal pain improvement compared to those without such treatment; however, this association did not reach statistical significance. Dosage was not included in the analysis because of the high variability in therapeutic regimens, which made it difficult to establish sufficiently robust groups for meaningful comparison. No other sociodemographic or clinical variables showed significant differences between the two groups, suggesting that initial symptom intensity is the most reliable indicator of long-term gabapentinoid effectiveness in this setting.

Table 5 shows the results of the multivariate logistic regression analysis. In the model, we included Maximal improvement as the dependent variable. Covariables (potential confounders) tested in the unadjusted model (enter method) were male gender, age, presence of diabetes mellitus, presence of systemic arterial hypertension, DN4 score (baseline), VAS score (baseline) and the usage of antidepressants. The risk model showed significant relations between both VAS score (OR = 0.38, 95% CI: 0.20, 0.71, *p* = 0.003) and DN4 score (OR = 0.29, 95% CI: 0.14, 0.59, *p* ≤ 0.001) and antidepressants usage (OR = 3.01, 95% CI: 1.01, 8.92, *p* = 0.046), whereas no statistical associations were observed with the rest of covariables. In the second analysis, adjusting these potential confounders using the stepwise method, in the model, the same three variables remained significantly associated with maximal improvement: VAS score (OR = 0.43, 95% CI: 0.24, 0.76, *p* = 0.004) and DN4 score (OR = 0.33, 95% CI: 0.17, 0.65, *p* = 0.001) and antidepressants usage (OR = 3.33, 95% CI: 1.16, 9.56, *p* = 0.025).

### 3.5. Time-to-Event Analysis: Kaplan–Meier Estimates of Pain Improvement

**Figure 2:**
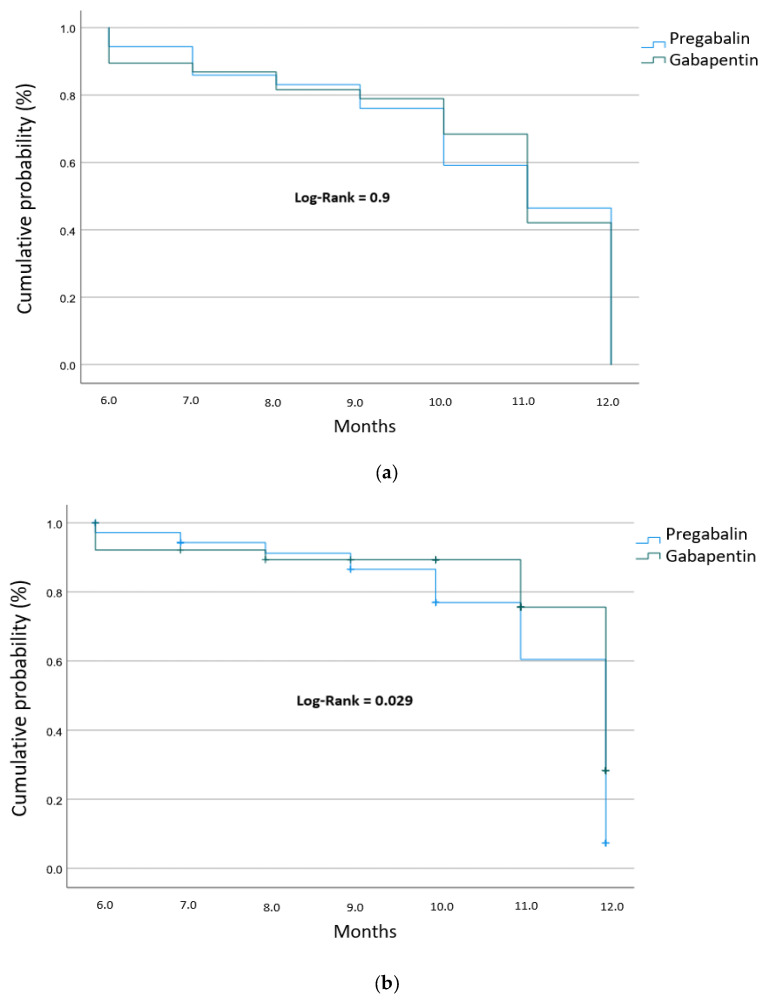
Kaplan–Meier survival curves showing the cumulative probability of pain improvement: (**a**) Time to improvement based on DN4 test scores; (**b**) Time to improvement based on VAS scores.

The cumulative probability of achieving clinical improvement over time was evaluated using Kaplan–Meier survival curves. Figure 2a illustrates the time-to-pain improvement based on the DN4 test criteria. No statistically significant differences were observed between patients treated with gabapentin versus those receiving pregabalin (*p* > 0.05), suggesting that both agents are equally effective in modulating the sensory characteristics of neuropathic pain over the follow-up period. In contrast, the analysis of pain intensity via the Visual Analog Scale (VAS) revealed significant differences between treatment groups (Figure 2b). Patients treated with pregabalin demonstrated a superior effectiveness profile in terms of time to recovery compared to those treated with gabapentin. Specifically, 20% of the pregabalin group achieved significant pain reduction by approximately month 10, whereas the gabapentin group reached the same threshold one month later. This difference in the rate of improvement was statistically significant (Log-rank test, *p* = 0.029), suggesting that pregabalin may offer a more rapid onset of clinical effectiveness in reducing overall pain intensity within this institutional setting.

### 3.6. Time for Maximal Improvement by Treatment Group

**Figure 3:**
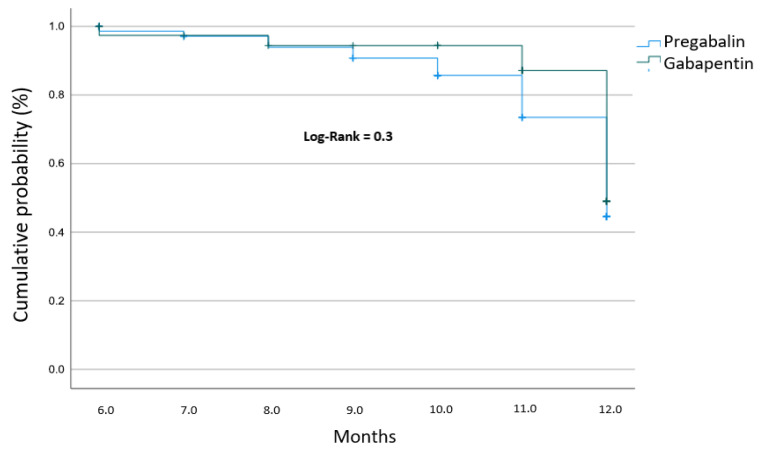
Kaplan–Meier survival curves for the time to achieve Maximal Improvement by gabapentinoid treatment group.

The cumulative probability of achieving the composite endpoint of Maximal Improvement (defined as DN4 = 1 and VAS ≤ 2) was further analyzed to compare the long-term effectiveness of both gabapentinoid agents. As shown in Figure 3, there was no statistically significant difference in the time to achieve maximal improvement between the gabapentin and pregabalin groups (Log-rank test, *p* = 0.30). Despite the faster initial reduction in pain intensity observed for pregabalin in the VAS-specific analysis, the long-term probability of reaching a state of minimal clinical symptoms was equivalent for both medications. This suggests that, while the kinetics of pain relief may differ between these two agents, their overall therapeutic ceiling for achieving complete clinical optimization is similar within this cohort.

### 3.7. Impact of Clinical and Demographic Risk Factors on Treatment Effectiveness

**Figure 4:**
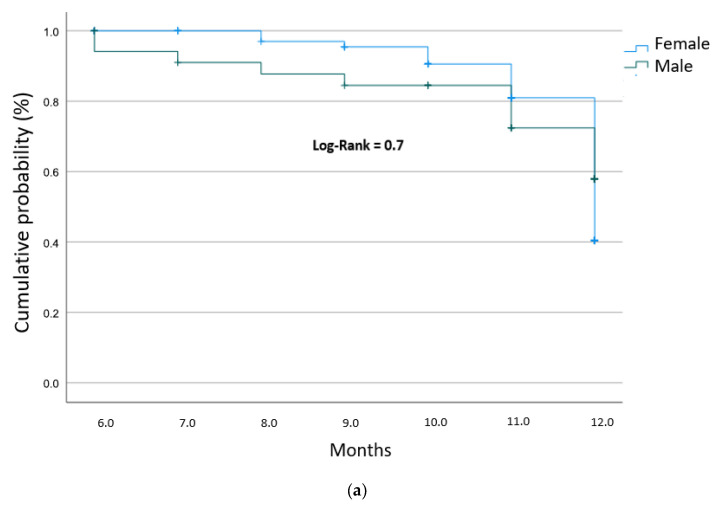

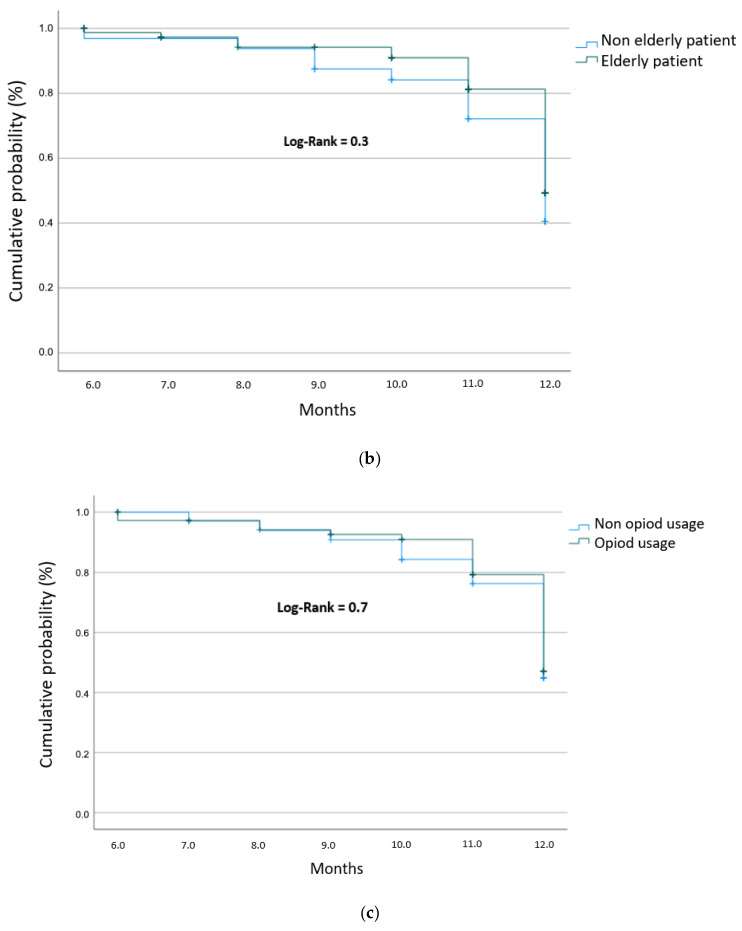

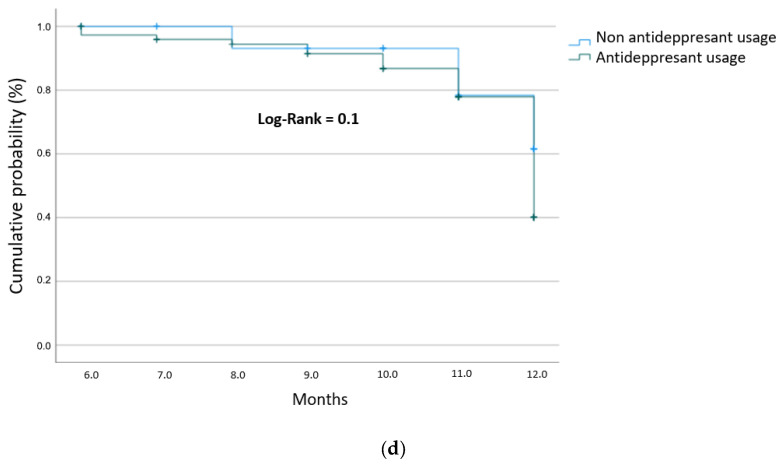
Kaplan–Meier survival curves comparing the time to maximal pain improvement stratified by: (**a**) Gender; (**b**) Age; (**c**) Concomitant opioid use; and (**d**) Concomitant antidepressant use.

To further explore the determinants of therapeutic success, a sub-analysis was performed using Kaplan–Meier curves to evaluate the influence of various risk factors on the time to achieve Maximal Improvement. The variables analyzed included gender (Figure 4a), elderly age (≥65 years; Figure 4b), concomitant opioid usage (Figure 4c), and the use of antidepressant agents (Figure 4d). The log-rank tests revealed no statistically significant differences in pain improvement across any of these subgroups (*p* > 0.05). Notably, despite the high pharmacological complexity and the advanced age of the cohort, the effectiveness of gabapentinoid therapy remained consistent.

## 4. Discussion

Gabapentinoid therapy effectively reduced both neuropathic-specific symptoms and general pain intensity in this clinical setting. By the end of the follow-up period, all 109 patients had experienced clinical improvement. Regarding neuropathic characteristics (DN4), both gabapentin and pregabalin showed comparable times to therapeutic improvement, achieving a 60% success rate at 12 months. This outcome aligns with large-scale meta-analyses positioning gabapentinoids as highly effective agents for various neuropathic etiologies, epilepsy, and fibromyalgia [11,12]. However, pregabalin demonstrated a faster onset of action for overall pain intensity (VAS), reaching clinical milestones one month earlier than gabapentin. Despite these kinetic differences, “Maximal Improvement” was achieved by only 35.8% of the cohort. This suggests that, while these agents are potent, complete symptomatic resolution remains challenging in complex clinical cases.

Our results align with those of Yilmaz et al. [13], who observed significant pain reduction after six months of gabapentinoid therapy in an outpatient setting. In contrast, while Vaishnav et al. [14] suggested that pregabalin is superior to gabapentin based on the DN4 test, our one-year longitudinal data show that these differences equilibrate over time. This discrepancy may be explained by the study duration. Short-term assessments often capture the faster titration, higher absorption rate, and greater bioavailability of pregabalin [12]. However, long-term follow-up—as observed in our study and supported by Dragic et al. [15]—reveals a similar therapeutic ceiling for both agents.

A key finding regarding medication safety is that all patients (100%) reported at least one adverse drug event (ADE), with somnolence being the most prevalent (66.1%). Furthermore, the frequent co-administration of gabapentinoids with opioids and antidepressants necessitates rigorous monitoring for synergistic adverse effects. Such combinations significantly increase the risk of profound sedation and respiratory depression, requiring a proactive pharmacovigilance approach in this high-risk population. This incidence is notably higher than that often reported in controlled trials. However, it remains consistent with the pharmacological mechanism of calcium channel modulation and the high-risk profile of elderly patients. In this population, these medications are frequently categorized as potentially inappropriate under specific clinical criteria [16,17]. While Muanda et al. [18] highlighted severe risks such as respiratory depression and encephalopathy in elderly populations, our cohort experienced no serious ADEs (SAEs) or hospitalizations.

The favorable safety outcomes, despite a high incidence of mild-to-moderate effects, may be attributed to the specialized monitoring provided within our institution. However, the high prevalence of concomitant opioid (66.1%) and antidepressant (67.9%) use in our sample creates a high-risk environment for synergistic CNS depression. Previous studies have warned that combining gabapentinoids with opioids significantly increases the risk of respiratory-related mortality [19]. Furthermore, the potential for misuse and dependence requires careful monitoring during long-term regimens [20]. These findings emphasize that “real-world” gabapentinoid safety is intrinsically linked to both the management of polypharmacy and the clinical complexity of the patient.

Several limitations of this study should be acknowledged. First, the retrospective nature of the data limited the analysis, as certain key variables were not captured. These include lifestyle factors, which are known to influence pain levels, and renal function metrics (e.g., eGFR or creatinine), which are critical for assessing gabapentinoid clearance. Furthermore, the generalizability of our findings is constrained because the study was conducted at a single center.

Additionally, the lack of standardized algorithms to establish causality for adverse events is a notable limitation. Because these events were retrospectively extracted from Electronic Health Records, associations can only be inferred based on the medication’s established safety profile. Finally, as the study was conducted within a highly specialized institution, the results may not be generalizable to primary care or general outpatient settings.

## 5. Conclusions

Gabapentinoid therapy is associated with clinically significant improvements in neuropathic pain; approximately 60% of patients achieved meaningful relief over a 12-month period. However, “Maximal Improvement” occurred in only one-third of the cohort. The universal incidence of adverse events—predominantly somnolence—underscores the need for proactive pharmacovigilance, particularly in patients with multimorbidity and polypharmacy. Our findings emphasize a patient-centered, multidisciplinary approach to balance analgesic efficacy against the side-effect burden in real-world practice. Furthermore, the clinical benefit of these agents appears resilient to factors such as concomitant opioid use or age-related physiological changes, reinforcing their role as versatile first-line treatments in specialized settings.

## Figures and Tables

**Table 1:** Sociodemographic and clinical characteristics of patients with neuropathic pain.

Variable, n (%)	n = 109 (100.0)
Gender, n (%)	
Female	75 (68.8)
Male	34 (31.2)
Age (yrs.), mean ± SD	66.2 ± 15.3
Elderly patients, n (%)	77 (70.6)
Diagnostic, n (%)	
Trigeminal neuralgia	38 (34.9)
Post-shingles neuralgia	65 (59.6)
Diabetic polyneuropathy	6 (5.5)
Type of comorbidities, n (%)	
DM	64 (58.7)
SAH	61 (56.0)
Musculoskeletal	32 (29.4)
Insomnia	18 (16.5)
Anxiety	17 (15.6)
Depression	17 (15.6)
Cancer	13 (11.9)
Others *	13 (11.9)

* Other comorbidities present in our cohort were: venous insufficiency, anemia, Parkinson’s disease and Alzheimer’s disease. Qualitative variables are expressed as frequency and percentage, while quantitative variables are expressed as means and standard deviation (SD).

**Table 2:** Pharmacological treatment characteristics and baseline pain levels.

Variable, n (%)	n = 109 (100.0)
Neuropathic pain treatment, n (%)	
Pregabalin	71 (65.1)
Gabapentin	38 (34.9)
DN4 (baseline), mean ± SD	5.2 ± 0.7
Neuropathic pain presence, n (%)	109 (100.0)
VAS (baseline), mean ± SD	8.9 ± 0.8
Concomitant drugs	
Analgesics, n (%)	76 (69.7)
Antidepressants, n (%)	74 (67.9)
Opioids, n (%)	72 (66.1)
Antiepileptics, n (%)	37 (33.9)

Qualitative variables are expressed as frequency and percentage, while quantitative variables are expressed as means and standard deviation (SD).

**Table 3:** Longitudinal pain improvement and safety profile.

Variable, n (%)	n = 109 (100.0)
DN4 (last visit), mean ± SD	1.5 ± 0.5
VAS, mean ± SD	2.2 ± 0.8
Maximal improvement, n (%)	39 (35.8)
Adverse events, n (%)	
Somnolence	72 (66.1)
Nausea	45 (41.3)
Headache	29 (26.6)
Confusion	22 (20.2)
Generalized weakness	6 (5.5)

Qualitative variables are expressed as frequency and percentage, while quantitative variables are expressed as means and standard deviation (SD).

**Table 4:** Comparison of baseline characteristics between patients with and without maximal improvement.

Variable, n (%)	Without Maximal Improvement n = 70 (100.0)	Maximal Improvement n = 39 (100.0)	*p*
Female, n (%)	47 (67.1)	28 (71.8)	0.6
Elderly patient, n (%)	53 (75.7)	24 (61.5)	0.1
DM, n (%)	41 (58.6)	23 (59.0)	0.9
SAH, n (%)	40 (57.1)	21 (53.8)	0.7
Pregabalin, n (%)	43 (61.4)	28 (71.8)	0.2
Gabapentin, n (%)	27 (38.6)	11 (28.2)	0.2
Treatment length (months), mean ± SD	10.3 ± 2.0	10.7 ± 1.7	0.2
DN4 (baseline), mean ± SD	5.4 ± 0.7	4.8 ± 0.5	<0.001
VAS (baseline), mean ± SD	9.1 ± 0.8	8.5 ± 0.7	0.001
Somnolence, n (%)	47 (67.1)	25 (64.1)	0.7
Nausea, n (%)	30 (42.9)	15 (38.5)	0.6
Confusion, n (%)	12 (17.1)	10 (25.6)	0.2
Headache, n (%)	17 (24.3)	12 (30.8)	0.4
Antidepressants, n (%)	43 (61.4)	31 (79.5)	0.053
Opioids, n (%)	47 (67.1)	25 (64.1)	0.7
Analgesics, n (%)	51 (72.9)	25 (64.1)	0.3
Antiepileptics, n (%)	26 (37.1)	11 (28.2)	0.3

Comparisons between proportions were performed using chi-square test, and comparisons between means were performed using Student *t*-tests.

**Table 5:** Variables associated with presenting Maximal improvement in patients using Gabapentinoids.

	Maximal Improvement					
	Unadjusted			Adjusted		
	Enter Method			Stepwise Method		
	OR	95% CI	*p*-Value	aOR	95% CI	*p*-Value
Male gender	0.49	0.17–1.39	0.1	---	---	---
Age	0.97	0.93–1.01	0.08	---	---	---
Diabetes Mellitus type 2	2.04	0.62–6.69	0.2	---	---	---
Hypertension	0.67	0.21–2.11	0.5	---	---	---
VAS score (baseline)	0.38	0.20–0.71	0.003	0.43	0.24–0.76	0.004
DN4 score (baseline)	0.29	0.14–0.59	<0.001	0.33	0.17–0.65	0.001
Antidepressant usage	3.01	1.01–8.92	0.046	3.33	1.16–9.56	0.025

Abbreviation: OR: odds ratio, aOR: adjusted odds ratio, CI: confidence interval.

## Data Availability

The data presented in this study are available on request from the corresponding author. The data are not publicly available due to institutional privacy policies and ethical restrictions related to the protection of individual-level electronic medical records.

## Abbreviations

The following abbreviations are used in this manuscript:

DM	Diabetes Mellitus Type 2
DN4	Questionnaire for neuropathic pain
SAH	Systemic arterial hypertension
Yrs.	Years
VAS	Visual Analog Scale
